# Dr. J. B. Neff

**Published:** 1913-09

**Authors:** 


Dr. J. B. Neff of Port Colborne, Ont., licensed in 1878, for many years a subscriber to this Journal, and a man held in high esteem by his confreres and fellow citizens, died early this summer. We have been unable to obtain accurate biographic data.

